# Metabolic regulation by secreted phospholipase A_2_

**DOI:** 10.1186/s41232-016-0012-7

**Published:** 2016-05-21

**Authors:** Hiroyasu Sato, Yoshitaka Taketomi, Makoto Murakami

**Affiliations:** 1 Lipid Metabolism Project, The Tokyo Metropolitan Institute of Medical Science, 2-1-6 Kamikitazawa, Setagaya-ku, Tokyo, 156-8506 Japan; 2 AMED-CREST, Japan Agency for Medical Research and Development, Tokyo, 100-0004 Japan

**Keywords:** Fatty acid, Lipoprotein, Obesity, Phospholipid, Phospholipase A_2_

## Abstract

Within the phospholipase A_2_ (PLA_2_) superfamily that hydrolyzes phospholipids to yield fatty acids and lysophospholipids, the secreted PLA_2_ (sPLA_2_) enzymes comprise the largest family that contains 11 isoforms in mammals. Individual sPLA_2_s exhibit unique distributions and specific enzymatic properties, suggesting their distinct biological roles. While sPLA_2_s have long been implicated in inflammation and atherosclerosis, it has become evident that they are involved in diverse biological events through lipid mediator-dependent or mediator-independent processes in a given microenvironment. In recent years, new biological aspects of sPLA_2_s have been revealed using their transgenic and knockout mouse models in combination with mass spectrometric lipidomics to unveil their target substrates and products in vivo. In this review, we summarize our current knowledge of the roles of sPLA_2_s in metabolic disorders including obesity, hepatic steatosis, diabetes, insulin resistance, and adipose tissue inflammation.

## Background

**Fig. 1:**
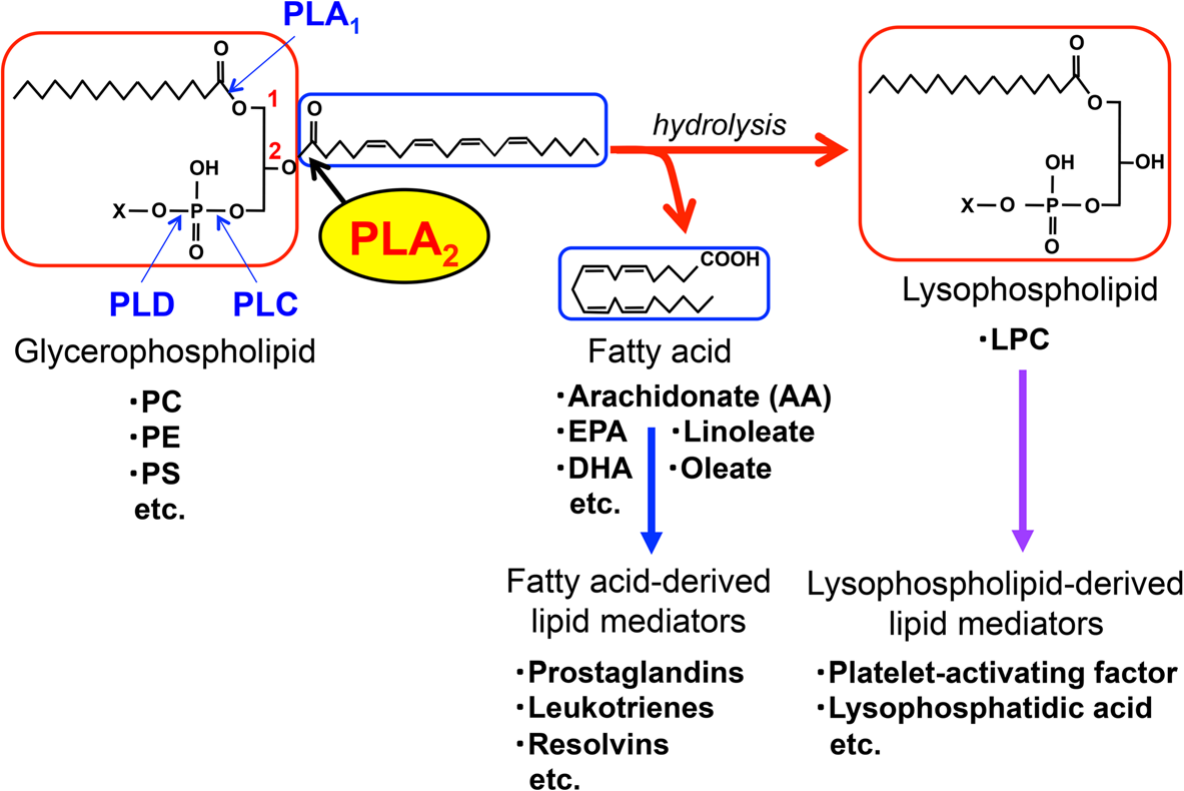
PLA_2_ reaction. PLA_2_ hydrolyzes the *sn*-2 position of glycerophospholipids to yield fatty acids (typically unsaturated) and lysophospholipids. Phospholipases A_1_, C, and D (PLA_1_, PLC, and PLD, respectively) cleave other ester bonds in the glycerophospholipid molecule. Unsaturated fatty acids and lysophospholipids are further metabolized to a variety of lipid mediators

Phospholipase A_2_ (PLA_2_) is a group of enzymes that hydrolyze phospholipids to yield fatty acids and lysophospholipids (Fig. 1). In general, this reaction is best known as the initial, rate-limiting step of arachidonate metabolism leading to the production of bioactive lipid mediators including prostaglandins and leukotrienes. The mammalian genome encodes more than 30 PLA_2_s or related enzymes, among which the secreted phospholipase A_2_ (sPLA_2_) family consists of low molecular mass and Ca^2+^-requiring enzymes with a conserved His-Asp catalytic dyad and includes 11 isoforms (IB, IIA, IIC, IID, IIE, IIF, III, V, X, XIIA, and XIIB) [1–5]. Beyond cytosolic PLA_2_ (cPLA_2_α; group IVA PLA_2_) whose regulatory roles in arachidonate metabolism have been well documented [6], the biological roles of sPLA_2_s remained a mystery for more than two decades. Recent studies using mice that have been gene manipulated for sPLA_2_s have begun to reveal their distinct and unique roles in various biological events [7–14]. The current understanding of the in vivo functions of sPLA_2_s has been summarized in several reviews [1–5].

Historically, sPLA_2_s have long been implicated in inflammation and atherosclerosis. This idea stems from the observations that sPLA_2_-IIA, a prototypic “inflammatory sPLA_2_,” is induced during inflammation [15] and that hydrolysis of low-density lipoprotein (LDL) by sPLA_2_s gives rise to pro-atherogenic LDL, which promotes macrophage foam cell formation in vitro [16, 17]. Indeed, subsequent genetic and pharmacological approaches support the pro-inflammatory or atherosclerotic roles of sPLA_2_s [10–14]. However, the regulatory roles of sPLA_2_s in metabolic disorders including obesity and insulin resistance have not yet been fully elucidated. Recently, it has become clear that several sPLA_2_s are expressed in the adipose tissue or gastrointestinal (GI) tract and have variable influences on systemic metabolic states [18–20]. Here, we will make an overview of the novel biological roles of sPLA_2_s and the lipid pathways underlying metabolic regulation, as revealed by sophisticated knockout and lipidomics techniques.

### sPLA_2_-V, a “metabolic sPLA_2_”

Metabolic syndrome is increasing at an explosive rate worldwide due to a pandemic of obesity associated with diabetes, insulin resistance, non-alcoholic fatty liver disease, and hyperlipidemia [21]. The mechanisms connecting obesity to insulin resistance include an elevated level of circulating lipids, ectopic lipid deposition leading to lipotoxicity, and chronic inflammation in metabolically active tissues [22]. Obesity arises through the dysregulations of intracellular lipid metabolism or extracellular lipid partitioning among tissues, and the perturbation of intracellular/extracellular lipases variably and often profoundly affect obesity and insulin resistance [23–26]. For instance, lipoprotein lipase is an obesity susceptibility factor showing an inverse relationship between its activity and obesity-related traits in humans [23]. The imbalanced accumulation of LDL in favor of high-density lipoprotein (HDL) is a critical risk factor not only for atherosclerosis but also for insulin intolerance [27]. As lipoprotein particles are shielded by phospholipids, aberrant lipoprotein phospholipid metabolism could also influence lipid partitioning and thereby obesity.

Among the sPLA_2_ isoforms, sPLA_2_-V potently hydrolyzes phospholipids in lipoproteins (LDL > HDL) in vitro [17]. However, studies using *Pla2g5* ^−/−^ mice have failed to demonstrate the participation of sPLA_2_-V in LDL metabolism in atherosclerosis models [11, 28]. Except for studies using sPLA_2_-overexpressing transgenic mice [17, 29, 30], no reports have firmly established whether endogenous sPLA_2_s affect lipoprotein metabolism in vivo. In a microarray search for unique lipase-related genes whose expressions are associated with obesity, we recently found that sPLA_2_-V (and sPLA_2_-IIE; see below) is robustly induced in adipocytes of obese mice [18]. This sPLA_2_-V induction is dependent on adipogenesis plus endoplasmic reticulum (ER) stress. Because of this property plus the fact that sPLA_2_-V is constitutively expressed at relatively high levels in several metabolic tissues such as the heart and skeletal muscle, we refer to sPLA_2_-V as a “metabolic sPLA_2_.”

Notably, when fed a high-fat diet (HFD), *Pla2g5* ^*−/−*^ mice display hyperlipidemia with higher plasma levels of LDL, increased obesity and hepatic steatosis, and lower insulin sensitivity [18]. Furthermore, the adipose tissues in *Pla2g5* ^*−/−*^ mice show a greater infiltration of M1 macrophages and a higher expression of pro-inflammatory cytokines. Thus, sPLA_2_-V plays anti-obesity and anti-inflammatory roles in the context of metabolic disorders. Lipidomics have revealed that sPLA_2_-V secreted from hypertrophic adipocytes preferentially hydrolyzes phosphatidylcholine (PC) in fat-overladen LDL to release unsaturated fatty acids (e.g., oleate and linoleate) in vivo [18]. As such, the increased LDL lipid levels in *Pla2g5* ^−/−^ mice could impact on adipocyte hypertrophy and the fatty liver. Furthermore, in accordance with the alterations in LDL phospholipids, the levels of free oleate and linoleate are lower in the adipose tissue of HFD-fed *Pla2g5* ^*−/−*^ mice than in that of WT mice. These unsaturated fatty acids released by sPLA_2_-V dampen the M1 macrophage polarization by saturated fatty acids (e.g., palmitate) likely through the attenuation of ER stress. This mechanism fits with the view that sPLA_2_-V has an apparent, even if not strict, substrate preference for PC bearing a fatty acid with a low degree of unsaturation.

**Fig. 2:**
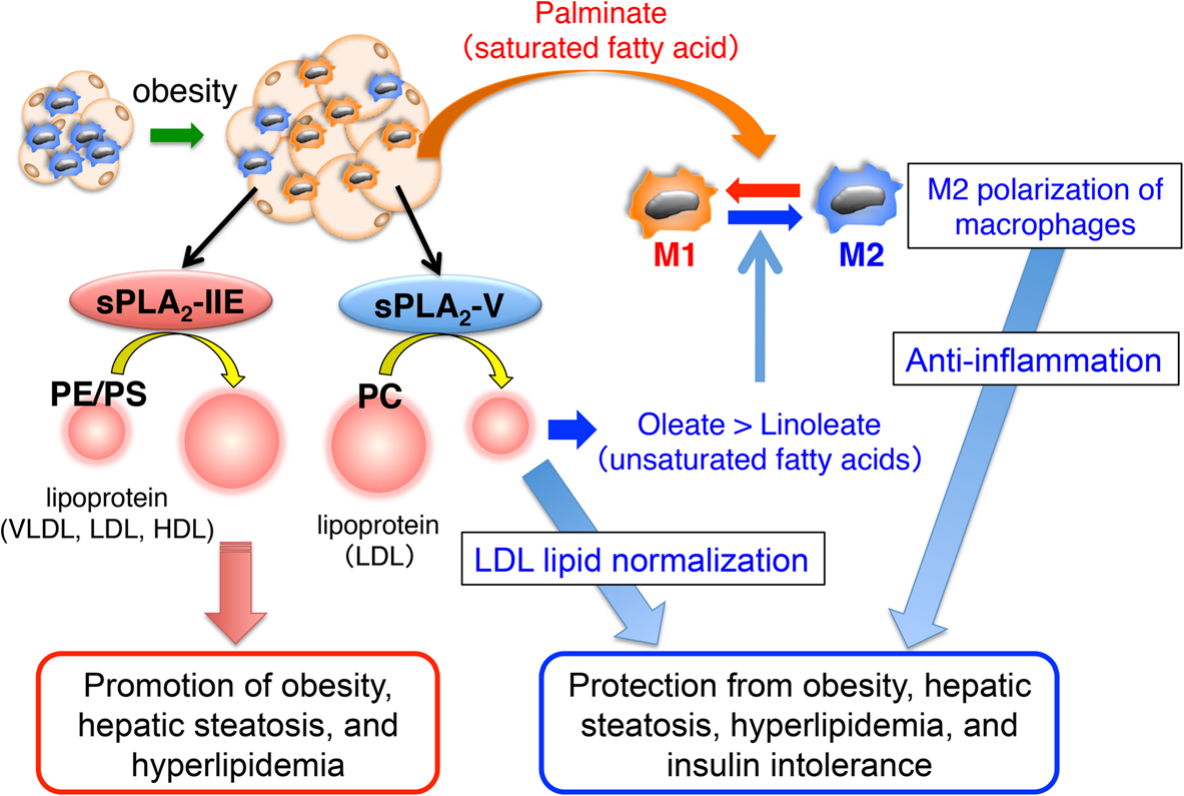
Metabolic regulation by “metabolic sPLA_2_s.” During obesity, two sPLA_2_s (IIE and V) are induced in hypertrophic adipocytes. sPLA_2_-IIE hydrolyzes PE and PS in lipoproteins (VLDL, LDL, and HDL) and facilitates fat accumulation into the peripheral tissues. sPLA_2_-V hydrolyzes PC in LDL to release oleate and linoleate, which counteracts the palmitate-induced M1 polarization of macrophages and thereby sequesters adipose tissue inflammation [18]

It remains obscure whether the sPLA_2_-V action would depend on the production of ω6 arachidonic acid-derived eicosanoids (e.g., prostaglandins and leukotrienes) or ω3 polyunsaturated fatty acid (e.g., eicosapentaenoic acid and docosahexaenoic acid)-derived pro-resolving lipid mediators (e.g., resolvins and protectins), since the adipose tissue levels of these fatty acid metabolites were not affected by *Pla2g5* deficiency. Rather, sPLA_2_-V contributes to controlling the quality of the lipids, i.e., the balance between saturated (detrimental) and unsaturated (beneficial) fatty acids, in adipose tissue microenvironments, providing a novel insight into the sPLA_2_ action beyond lipid mediators. Together, these results reveal a functional link between lipoprotein metabolism and anti-inflammation for this particular sPLA_2_ and provide a rationale for the long-standing issue of the physiological importance of lipoprotein hydrolysis by this extracellular enzyme family (Fig. 2).

**Fig. 3:**
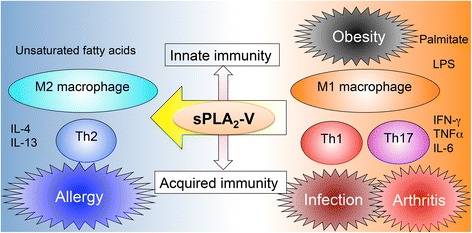
Immune balance regulation by sPLA_2_-V. sPLA_2_-V is induced in the M2 macrophages and Th2 cells by IL-4 or IL-13 and promotes Th2/M2-dominant immunity such as asthma [31, 32]. Conversely, sPLA_2_-V plays protective roles in Th1- or Th17-type immune responses including obesity, infection, and arthritis [10, 18, 34]

Another intriguing feature of sPLA_2_-V is that it is a “Th2/M2-prone sPLA_2_,” allowing a shift in the immune balance toward the Th2/M2 status. Apart from the crucial role of adipocyte- rather than macrophage-derived sPLA_2_-V in obesity, the *Pla2g5* expression in macrophages is markedly induced by the M2-skewing Th2 cytokines IL-4 and IL-13 and the *Pla2g5* ablation decreases the Th2-mediated immune responses [18, 31]. In vitro*,* exogenous sPLA_2_-V is capable of facilitating the M2 polarization of macrophages probably through augmenting the prostaglandin E_2_ production [18]. Furthermore, in human macrophages, sPLA_2_-V induced by IL-4 promotes phagocytosis through the production of lysophosphatidylethanolamine [32]. Given the increased incidence of metabolic disorders resulting from the genetic ablation of Th2 or M2 inducers (e.g., *Il4*, *Il13*, *Il33*, *Stat6*, or *Pparg*) [33], the decreased whole-body Th2/M2 status resulting from *Pla2g5* deficiency may also contribute to the exacerbation of obesity-associated inflammation. This notion also accords with the observations that *Pla2g5* ^−/−^ mice are protected from asthma (Th2 dependent) [31], while suffering from more severe fungal infection (Th1 dependent) or arthritis (Th17 dependent) [10, 34], where the Th2 immunity counteracts the Th1/Th17-based inflammations. Thus, the fact that sPLA_2_-V acts as a Th2/M2-prone sPLA_2_ can account for the pro- versus anti-inflammatory actions of this enzyme in distinct immunopathological settings (Fig. 3).

Notably, in humans, *Pla2g5* gene polymorphisms correlate with the LDL levels in subjects with type 2 diabetes or obesity [35, 36]. The in vitro sPLA_2_-V susceptibility of LDL from patients with type 2 diabetes is greater than that of LDL from healthy controls [37]. Moreover, the *Pla2g5* expression in the human visceral adipose tissue inversely correlates with LDL plasma levels [18]. These results imply a human relevance for the metabolic role of sPLA_2_-V.

### sPLA_2_-IIE, another “metabolic sPLA_2_”

We also found that sPLA_2_-IIE, which remained a functionally orphan sPLA_2_ for more than a decade, acts as another “metabolic” sPLA_2_ that is induced in hypertrophic adipocytes [18]. An adipogenic stimulus is sufficient for induction of sPLA_2_-IIE in adipocytes. *Pla2g2e* ^−/−^ mice are modestly protected from diet-induced obesity, hepatic steatosis, and hyperlipidemia. In contrast to sPLA_2_-V, which hydrolyzes PC in LDL to selectively release oleate and linoleate (see above), sPLA_2_-IIE preferentially hydrolyzes minor lipoprotein phospholipids, phosphatidylserine (PS), and phosphatidylethanolamine (PE), with no apparent fatty acid selectivity. As such, sPLA_2_-IIE alters the lipid composition of lipoproteins, thereby moderately affecting the lipid accumulation in the adipose tissue and liver.

Although the molecular mechanism that links lipoprotein PS/PE hydrolysis with obesity still remains unclear, this study revealed for the first time the importance of these minor lipoprotein phospholipids in metabolic regulation. As the increase of the negative charges in lipoproteins by oxidative modification renders the particles smaller, the increase of the anionic phospholipids (e.g., PS) in lipoproteins by the absence of sPLA_2_-IIE may also afford a similar effect. Alternatively, lysophosphatidylethanolamine or lysophosphatidylserine produced by sPLA_2_-IIE might have some metabolic effects, a possibility that awaits future studies. Collectively, these results underscore the physiological relevance of lipoprotein hydrolysis by distinct sPLA_2_s and highlight the importance of “metabolic sPLA_2_s” as the integrated regulators of metabolic responses (Fig. 2).

On the other hand, another study has recently reported that *Pla2g2e* ^−/−^ mice accumulate more epididymal fat as they age [38]. During adipogenesis, the genetic deletion or siRNA knockdown of sPLA_2_-IIE increases the triglyceride in adipocytes, while its overexpression or exogenous addition facilitates lipolysis. Although the reason for the discrepancy between the two studies is unclear, it might have arisen from different experimental conditions (HFD versus chow diets or female versus male mice) in different animal facilities.

### sPLA_2_-IB, a “digestive sPLA_2_”

Systemic lipid metabolism is often affected by the digestion and absorption of dietary lipids in the GI tract. sPLA_2_-IB is synthesized by pancreatic acinar cells, and after secretion as a zymogen into pancreatic juice, an *N*-terminal propeptide of the inactive zymogen is cleaved by trypsin to yield an active enzyme in the duodenum. The hydrolysis of PC by sPLA_2_-IB is greatly accelerated in the presence of a low concentration of detergent such as deoxycholate [39]. This property appears to be physiologically important since the digestion of dietary phospholipids by sPLA_2_-IB occurs in the presence of bile acid in the GI tract.

*Pla2g1b* ^−/−^ mice show resistance to obesity, lower plasma insulin and leptin levels, and improved glucose tolerance when fed a high-fat/carbohydrate diet [40]. These phenotypes of *Pla2g1b* ^−/−^ mice are most likely due to a marked reduction in the hydrolysis of dietary and biliary PC and thereby in the production and absorption of lysophosphatidylcholine (LPC) in the GI tract. The increased intestinal absorption of LPC promotes postprandial hyperglycemia by inhibiting the glucose uptake by the liver and muscle, and accordingly, the absence of sPLA_2_-IB reduces the postprandial LPC levels, leading to improved insulin sensitivity and hepatic fatty acid oxidation [41, 42]. It is noteworthy that *Pla2g1b* ^−/−^ mice on a *Ldlr* ^*−/−*^ background are protected from body weight gain and atherosclerosis in response to a hypercaloric diet [43] and that the oral administration of the sPLA_2_ inhibitor methyl indoxam along with a diabetogenic diet effectively suppresses diet-induced obesity and diabetes in mice likely through the prevention of the intestinal digestion of dietary and biliary PC by sPLA_2_-IB [44]. In further support of these observations, pancreatic acinar cell-specific *Pla2g1b*-transgenic mice develop more severe obesity and insulin resistance [45]. These results suggest that the inhibition of sPLA_2_-IB, a “digestive sPLA_2_,” may be an effective oral therapeutic option for the treatment of diet-induced obesity and diabetes.

### Complex and enigmatic roles of sPLA_2_-X in metabolism

Lastly, we briefly summarize the possible metabolic roles of sPLA_2_-X, although details remain uncertain because of the fact that conflicting results have been obtained. Like sPLA_2_-IB, sPLA_2_-X also has an *N*-terminal propeptide, and its proteolytic removal leads to the full activation of the enzyme. A series of studies have provided some insights into the functional link of sPLA_2_-X-released polyunsaturated fatty acids to lipid-sensing nuclear receptor signaling. Macrophages from *Pla2g10* ^−/−^ mice show an increased expression of the cholesterol efflux transporters ABCA1 and ABCG1, and this effect appears to be dependent on the suppression of the liver X receptor (LXR) by sPLA_2_-X-released polyunsaturated fatty acids [46]. Moreover, the increased cholesterol content of the lipid rafts in *Pla2g10* ^−/−^ macrophages leads to significant reduction of endotoxin-induced inflammation [47]. The sPLA_2_-X-dependent suppression of LXR can also occur in the adipose tissue, where *Pla2g10* deficiency facilitates adipogenesis and obesity [48], and in the adrenal glands, where its deficiency promotes corticosteroidogenesis through the activation of steroidogenic acute regulatory protein [49]. In the latter case, pro-sPLA_2_-X is proteolytically processed to a mature, active form by the protein convertases furin and PCSK6, which are induced by the adrenocorticotropic hormone, in adrenal cells [50]. However, as far as we have been able to examine, the *Pla2g10* expression in mouse macrophages and the adipose tissue is very low, arguing against the above observations. Rather, we prefer the idea that sPLA_2_-X might be expressed in a limited subset of these cells or supplied from proximal or distal cells in a paracrine manner.

On the other hand, we have shown that sPLA_2_-X is expressed abundantly in GI-lining cells and participates in phospholipid digestion [19]. Accordingly, *Pla2g10* ^−/−^ mice display a reduced age-associated adiposity and improved insulin sensitivity in the skeletal muscle, likely through a mechanism reminiscent of that in *Pla2g1b* ^−/−^ mice. Thus, the two “digestive sPLA_2_s” (IB and X) may spatiotemporally control the hydrolysis of dietary and biliary phospholipids and thereby the absorption of their hydrolytic products, depending on the quantity and quality of the dietary and biliary fat input. As in the case of *Pla2g2e* ^−/−^ mice (see above), the opposite phenotypes of *Pla2g10* ^−/−^ mice observed in different studies might have been due to differences in the experimental models or housing conditions employed, and further studies will be necessary to clarify more definitively the roles and mechanistic actions of sPLA_2_-X in metabolism.

It has been recently reported that the glucose-stimulated insulin secretion by islet β cells is augmented in *Pla2g10* ^−/−^ mice, underscoring a novel metabolic role of sPLA_2_-X [51]. Mechanistically, sPLA_2_-X negatively regulates insulin secretion by augmenting the cyclooxygenase-2-dependent prostaglandin E_2_ production. In this scenario, targeting sPLA_2_-X may be an effective therapeutic option for enhancing β cell function in the treatment of diabetes.

## Conclusions

It is now obvious that at least four sPLA_2_s are involved in metabolic regulation through distinct mechanisms, as summarized below. sPLA_2_-V is induced in hypertrophic adipocytes by obesity-associated ER stress and hydrolyzes PC in hyperlipidemic LDL to facilitate the skewing of macrophages from M1 to M2 subsets, thereby conferring protection from adipose tissue inflammation, insulin resistance, obesity, hepatic steatosis, and hyperlipidemia. The saturated fatty acids supplied abundantly from adipocytes trigger the M1 polarization of macrophages, which is counterregulated by the sPLA_2_-V-driven unsaturated fatty acids from LDL. sPLA_2_-IIE is induced in adipocytes in accordance with adipogenesis and hydrolyzes PE and PS in lipoproteins, eventually promoting fat storage in the adipose tissue and liver. sPLA_2_-IB, a pancreatic sPLA_2_ that is secreted into the GI lumen, hydrolyzes dietary and biliary phospholipids to promote lipid digestion and absorption, which is associated with obesity and hepatic insulin resistance. sPLA_2_-X variably affects metabolism possibly through the production of polyunsaturated fatty acids that modify the LXR signaling in the adipose tissue, through the digestion of the dietary and biliary phospholipids in the gut, or through the generation of prostaglandin E_2_ that suppresses insulin secretion in the pancreatic islet. In addition, sPLA_2_-IIA is abundantly expressed in the human and rat adipose tissues in obesity and the pharmacological inhibition of this isoform attenuates the adipose tissue inflammation in rats [18, 52]. It remains possible that other sPLA_2_ isoforms may also participate in metabolic regulation, and this issue is now under investigation. Together, these studies have brought about a paradigm shift toward a better understanding of the biological roles of this extracellular lipolytic enzyme family as coordinators of metabolism.

## References

[1] Lambeau G, Gelb MH (2008). Biochemistry and physiology of mammalian secreted phospholipases A_2_. Annu Rev Biochem.

[2] Murakami M, Taketomi Y, Girard C, Yamamoto K, Lambeau G (2010). Emerging roles of secreted phospholipase A_2_ enzymes: lessons from transgenic and knockout mice. Biochimie.

[3] Dennis EA, Cao J, Hsu YH, Magrioti V, Kokotos G (2011). Phospholipase A_2_ enzymes: physical structure, biological function, disease implication, chemical inhibition, and therapeutic intervention. Chem Rev.

[4] Murakami M, Taketomi Y, Miki Y, Sato H, Hirabayashi T, Yamamoto K (2011). Recent progress in phospholipase A_2_ research: from cells to animals to humans. Prog Lipid Res.

[5] Murakami M, Sato H, Miki Y, Yamamoto K, Taketomi Y (2015). A new era of secreted phospholipase A_2_. J Lipid Res.

[6] Uozumi N, Kume K, Nagase T, Nakatani N, Ishii S, Tashiro F (1997). Role of cytosolic phospholipase A_2_ in allergic response and parturition. Nature.

[7] Taketomi Y, Ueno N, Kojima T, Sato H, Murase R, Yamamoto K (2013). Mast cell maturation is driven via a group III phospholipase A_2_-prostaglandin D_2_-DP1 receptor paracrine axis. Nat Immunol.

[8] Sato H, Taketomi Y, Isogai Y, Miki Y, Yamamoto K, Masuda S (2010). Group III secreted phospholipase A_2_ regulates epididymal sperm maturation and fertility in mice. J Clin Invest.

[9] Yamamoto K, Taketomi Y, Isogai Y, Miki Y, Sato H, Masuda S (2011). Hair follicular expression and function of group X secreted phospholipase A_2_ in mouse skin. J Biol Chem.

[10] Boilard E, Lai Y, Larabee K, Balestrieri B, Ghomashchi F, Fujioka D (2010). A novel anti-inflammatory role for secretory phospholipase A_2_ in immune complex-mediated arthritis. EMBO Mol Med.

[11] Bostrom MA, Boyanovsky BB, Jordan CT, Wadsworth MP, Taatjes DJ, de Beer FC (2007). Group V secretory phospholipase A_2_ promotes atherosclerosis: evidence from genetically altered mice. Arterioscler Thromb Vasc Biol.

[12] Henderson WR, Chi EY, Bollinger JG, Tien YT, Ye X, Castelli L (2007). Importance of group X-secreted phospholipase A_2_ in allergen-induced airway inflammation and remodeling in a mouse asthma model. J Exp Med.

[13] Muñoz NM, Meliton AY, Arm JP, Bonventre JV, Cho W, Leff AR (2007). Deletion of secretory group V phospholipase A_2_ attenuates cell migration and airway hyperresponsiveness in immunosensitized mice. J Immunol.

[14] Yamamoto K, Miki Y, Sato M, Taketomi Y, Nishito Y, Taya C (2015). The role of group IIF-secreted phospholipase A2 in epidermal homeostasis and hyperplasia. J Exp Med.

[15] Seilhamer JJ, Pruzanski W, Vadas P, Plant S, Miller JA, Kloss J (1989). Cloning and recombinant expression of phospholipase A_2_ present in rheumatoid arthritic synovial fluid. J Biol Chem.

[16] Hanasaki K, Yamada K, Yamamoto S, Ishimoto Y, Saiga A, Ono T (2002). Potent modification of low density lipoprotein by group X secretory phospholipase A_2_ is linked to macrophage foam cell formation. J Biol Chem.

[17] Sato H, Kato R, Isogai Y, Saka G, Ohtsuki M, Taketomi Y (2008). Analyses of group III secreted phospholipase A_2_ transgenic mice reveal potential participation of this enzyme in plasma lipoprotein modification, macrophage foam cell formation, and atherosclerosis. J Biol Chem.

[18] Sato H, Taketomi Y, Ushida A, Isogai Y, Kojima T, Hirabayashi T (2014). The adipocyte-inducible secreted phospholipases PLA2G5 and PLA2G2E play distinct roles in obesity. Cell Metab.

[19] Sato H, Isogai Y, Masuda S, Taketomi Y, Miki Y, Kamei D (2011). Physiological roles of group X-secreted phospholipase A_2_ in reproduction, gastrointestinal phospholipid digestion, and neuronal function. J Biol Chem.

[20] Hui DY (2012). Phospholipase A_2_ enzymes in metabolic and cardiovascular diseases. Curr Opin Lipidol.

[21] Despres JP, Lemieux I (2006). Abdominal obesity and metabolic syndrome. Nature.

[22] Hotamisligil GS (2006). Inflammation and metabolic disorders. Nature.

[23] Chen Y, Zhu J, Lum PY, Yang X, Pinto S, MacNeil DJ (2008). Variations in DNA elucidate molecular networks that cause disease. Nature.

[24] Chiu HK, Qian K, Ogimoto K, Morton GJ, Wisse BE, Agrawal N (2010). Mice lacking hepatic lipase are lean and protected against diet-induced obesity and hepatic steatosis. Endocrinology.

[25] Haemmerle G, Lass A, Zimmermann R, Gorkiewicz G, Meyer C, Rozman J (2006). Defective lipolysis and altered energy metabolism in mice lacking adipose triglyceride lipase. Science.

[26] Wang H, Knaub LA, Jensen DR, Young Jung D, Hong EG, Ko HJ (2009). Skeletal muscle-specific deletion of lipoprotein lipase enhances insulin signaling in skeletal muscle but causes insulin resistance in liver and other tissues. Diabetes.

[27] Avramoglu RK, Basciano H, Adeli K (2006). Lipid and lipoprotein dysregulation in insulin resistant states. Clin Chim Acta.

[28] Boyanovsky B, Zack M, Forrest K, Webb NR (2009). The capacity of group V sPLA_2_ to increase atherogenicity of ApoE^−/−^ and LDLR^−/−^ mouse LDL in vitro predicts its atherogenic role in vivo. Arterioscler Thromb Vasc Biol.

[29] Ivandic B, Castellani LW, Wang XP, Qiao JH, Mehrabian M, Navab M (1999). Role of group II secretory phospholipase A_2_ in atherosclerosis: 1. Increased atherogenesis and altered lipoproteins in transgenic mice expressing group IIa phospholipase A_2_. Arterioscler Thromb Vasc Biol.

[30] Yamamoto K, Isogai Y, Sato H, Taketomi Y, Murakami M (2011). Secreted phospholipase A_2_, lipoprotein hydrolysis, and atherosclerosis: integration with lipidomics. Anal Bioanal Chem.

[31] Ohta S, Imamura M, Xing W, Boyce JA, Balestrieri B (2013). Group V secretory phospholipase A_2_ is involved in macrophage activation and is sufficient for macrophage effector functions in allergic pulmonary inflammation. J Immunol.

[32] Rubio JM, Rodríguez JP, Gil-de-Gómez L, Guijas C, Balboa MA, Balsinde J (2015). Group V secreted phospholipase A_2_ is upregulated by IL-4 in human macrophages and mediates phagocytosis via hydrolysis of ethanolamine phospholipids. J Immunol.

[33] Odegaard JI, Chawla A (2013). The immune system as a sensor of the metabolic state. Immunity.

[34] Balestrieri B, Maekawa A, Xing W, Gelb MH, Katz HR, Arm JP (2009). Group V secretory phospholipase A_2_ modulates phagosome maturation and regulates the innate immune response against Candida albicans. J Immunol.

[35] Sergouniotis PI, Davidson AE, Mackay DS, Lenassi E, Li Z, Robson AG (2011). Biallelic mutations in PLA2G5, encoding group V phospholipase A_2_, cause benign fleck retina. Am J Hum Genet.

[36] Wootton PT, Arora NL, Drenos F, Thompson SR, Cooper JA, Stephens JW (2007). Tagging SNP haplotype analysis of the secretory PLA_2_-V gene, PLA2G5, shows strong association with LDL and oxLDL levels, suggesting functional distinction from sPLA_2_-IIA: results from the UDACS study. Hum Mol Genet.

[37] Pettersson C, Fogelstrand L, Rosengren B, Stahlman S, Hurt-Camejo E, Fagerberg B (2008). Increased lipolysis by secretory phospholipase A_2_ group V of lipoproteins in diabetic dyslipidaemia. J Intern Med.

[38] Zhi H, Qu L, Wu F, Chen L, Tao J (2015). Group IIE secretory phospholipase A_2_ regulates lipolysis in adipocytes. Obesity (Silver Spring).

[39] Jain MK, Egmond MR, Verheij HM, Apitz-Castro R, Dijkman R, De Haas GH (1982). Interaction of phospholipase A_2_ and phospholipid bilayers. Biochim Biophys Acta.

[40] Huggins KW, Boileau AC, Hui DY (2002). Protection against diet-induced obesity and obesity-related insulin resistance in Group 1B PLA_2_-deficient mice. Am J Physiol Endocrinol Metab.

[41] Labonté ED, Kirby RJ, Schildmeyer NM, Cannon AM, Huggins KW, Hui DY (2006). Group 1B phospholipase A_2_-mediated lysophospholipid absorption directly contributes to postprandial hyperglycemia. Diabetes.

[42] Labonté ED, Pfluger PT, Cash JG, Kuhel DG, Roja JC, Magness DP (2010). Postprandial lysophospholipid suppresses hepatic fatty acid oxidation: the molecular link between group 1B phospholipase A_2_ and diet-induced obesity. FASEB J.

[43] Hollie NI, Konaniah ES, Goodin C, Hui DY (2014). Group 1B phospholipase A_2_ inactivation suppresses atherosclerosis and metabolic diseases in LDL receptor-deficient mice. Atherosclerosis.

[44] Hui DY, Cope MJ, Labonté ED, Chang HT, Shao J, Goka E (2009). The phospholipase A_2_ inhibitor methyl indoxam suppresses diet-induced obesity and glucose intolerance in mice. Br J Pharmacol.

[45] Cash JG, Kuhel DG, Goodin C, Hui DY (2011). Pancreatic acinar cell-specific overexpression of group 1B phospholipase A_2_ exacerbates diet-induced obesity and insulin resistance in mice. Int J Obes (Lond).

[46] Shridas P, Bailey WM, Gizard F, Oslund RC, Gelb MH, Bruemmer D (2010). Group X secretory phospholipase A_2_ negatively regulates ABCA1 and ABCG1 expression and cholesterol efflux in macrophages. Arterioscler Thromb Vasc Biol.

[47] Shridas P, Bailey WM, Talbott KR, Oslund RC, Gelb MH, Webb NR (2011). Group X secretory phospholipase A_2_ enhances TLR4 signaling in macrophages. J Immunol.

[48] Li X, Shridas P, Forrest K, Bailey W, Webb NR (2010). Group X secretory phospholipase A_2_ negatively regulates adipogenesis in murine models. FASEB J.

[49] Shridas P, Bailey WM, Boyanovsky BB, Oslund RC, Gelb MH, Webb NR (2010). Group X secretory phospholipase A_2_ regulates the expression of steroidogenic acute regulatory protein (StAR) in mouse adrenal glands. J Biol Chem.

[50] Layne JD, Shridas P, Webb NR (2015). Ectopically expressed pro-group X secretory phospholipase A_2_ is proteolytically activated in mouse adrenal cells by furin-like proprotein convertases: implications for the regulation of adrenal steroidogenesis. J Biol Chem.

[51] Shridas P, Zahoor L, Forrest KJ, Layne JD, Webb NR (2014). Group X secretory phospholipase A_2_ regulates insulin secretion through a cyclooxygenase-2-dependent mechanism. J Biol Chem.

[52] Iyer A, Lim J, Poudyal H, Reid RC, Suen JY, Webster J (2012). An inhibitor of phospholipase A_2_ group IIA modulates adipocyte signaling and protects against diet-induced metabolic syndrome in rats. Diabetes.

